# Characterization of rare *ABCC8* variants identified in Spanish pulmonary arterial hypertension patients

**DOI:** 10.1038/s41598-020-72089-1

**Published:** 2020-09-15

**Authors:** Mauro Lago-Docampo, Jair Tenorio, Ignacio Hernández-González, Carmen Pérez-Olivares, Pilar Escribano-Subías, Guillermo Pousada, Adolfo Baloira, Miguel Arenas, Pablo Lapunzina, Diana Valverde

**Affiliations:** 1 CINBIO, Universidade de Vigo, Vigo, Spain; 2 Instituto de Investigación Sanitaria Galicia Sur, Hospital Álvaro Cunqueiro, Vigo, Spain; 3 Instituto de Genética Médica y Molecular (INGEMM), Hospital Universitario La Paz-IdiPaz, Universidad Autónoma de Madrid, Madrid, Spain; 4 Centro de Investigación Biomédica en Red de enfermedades Raras (CIBERER), Instituto de Salud Carlos III, Madrid, Spain; 5 ITHACA, European Reference Network on Rare Congenital Malformations and Rare Intellectual Disability, Brussels, Belgium; 6 Servicio de Cardiología, Hospital Universitario Río Hortega, Valladolid, Spain; 7 Centro de Investigación Biomédica en Red de Enfermedades Cardiovasculares (CIBERCV), Instituto de Salud Carlos III, Madrid, Spain; 8 Unidad Multidisciplinar de Hipertensión Pulmonar, Servicio de Cardiología, Hospital Universitario, 12 de Octubre, Madrid, Spain; 9 Servicio de Cardiología, Hospital 12 de Octubre, Madrid, Spain; 10 Servicio de Neumología, Complejo Hospitalario de Pontevedra, Pontevedra, Spain

**Keywords:** Mutation, Cardiovascular biology, Gene regulation

## Abstract

Pulmonary Arterial Hypertension (PAH) is a rare and fatal disease where knowledge about its genetic basis continues to increase. In this study, we used targeted panel sequencing in a cohort of 624 adult and pediatric patients from the Spanish PAH registry. We identified 11 rare variants in the ATP-binding Cassette subfamily C member 8 (*ABCC8*) gene, most of them with splicing alteration predictions. One patient also carried another variant in *SMAD1* gene (c.27delinsGTAAAG). We performed an *ABCC8 *in vitro biochemical analyses using hybrid minigenes to confirm the correct mRNA processing of 3 missense variants (c.211C > T p.His71Tyr, c.298G > A p.Glu100Lys and c.1429G > A p.Val477Met) and the skipping of exon 27 in the novel splicing variant c.3394G > A. Finally, we used structural protein information to further assess the pathogenicity of the variants. The results showed 11 novel changes in *ABCC8* and 1 in *SMAD1* present in PAH patients. After in silico and in vitro biochemical analyses, we classified 2 as pathogenic (c.3288_3289del and c.3394G > A), 6 as likely pathogenic (c.211C > T, c.1429G > A, c.1643C > T, c.2422C > A, c.2694 + 1G > A, c.3976G > A and *SMAD1* c.27delinsGTAAAG) and 3 as Variants of Uncertain Significance (c.298G > A, c.2176G > A and c.3238G > A). In all, we show that coupling in silico tools with in vitro biochemical studies can improve the classification of genetic variants.

## Introduction

Pulmonary Arterial Hypertension (PAH) is a rare form of Pulmonary Hypertension (PH, WHO group 1) characterized by the remodeling of the precapillary pulmonary arteries, producing an increase in blood flow resistance that eventually leads to right heart failure and death^1^. PAH is a rare disease (ORPHA #182090) that in Spain affects over 16 adults/million inhabitants, with an incidence of 3.7 cases/million inhabitants per year as shown by the Spanish PAH registry for patients over 18 years (REHAP)^2^.

PAH comprises a wide variety of clinical representations with different etiopathogenesis that share clinical and anatomopathological characteristics. PAH can be subdivided in different subtypes: idiopathic (IPAH), heritable (HPAH), induced by toxins or drugs, associated with other diseases (APAH) such as connective tissue disease, HIV infection and congenital heart disease among many others. PAH long term responders to calcium channel blockers, PAH with overt features of venous/capillaries (PVOD/PCH, often misdiagnosed as IPAH) involvement, and persistent PH of the newborn^3^.

The genetic basis of PAH has been slowly uncovered since the 2000s when mutations in the Bone Morphogenetic Protein Receptor of type 2 (*BMPR2*) where described^4,5^. Mutations in *BMPR2* are the most common cause of PAH, being present in up to a 70–80% of HPAH cases and 10–20% of IPAH^6^. More than 17 genes have been linked to the disease with different levels of evidence; with higher evidence: *BMPR2*, *EIF2AK4*, *TBX4*, *ATP13A3*, *GDF2*; *SOX17*, *AQP1*, *ACVRL1*, *SMAD9*, *ENG*, *KCNK3*, *CAV1*; with lesser: *SMAD4*, *SMAD1*, *KLF2*, *BMPR1B*, *KCNA5*^7,8^; and the newest addition *ABCC8*^9^. Mainly, PAH shows an autosomal dominant inheritance pattern with sex dependent incomplete penetrance, 14% in males and 42% in females^10^, this helps to understand why 60–80% of the patients in most of the registries are female^11,12^. Thus, additional factors influencing the development of the disease, as environmental, epigenetic and genetic background roles have not been clearly elucidated yet.

The identification of mutations in the ion channel coding genes *KCNK3* (TASK1) and *KCNA5* (K_v_1.5) first labeled PAH as a channelopathy^13,14^. Since their description, plenty of causal mutations have been described and tested in these genes^15–17^, and novel treatments for PAH have been proposed^18^. Alterations in genes coding for ATP related channels have also been described, such as *ATP1A2* (encoding for α2-subunit of the Na^+^/K^+^-ATPase)^19^, *ATP13A3*^7^, which codes for a member of the P-type ATPase family of proteins including *ABCC8*^9^.

*ABCC8* (ATP-binding Cassette subfamily C member 8) codifies for SUR1, a subunit of the hetero-octameric membrane K_ATP_ protein complexes. K_ATP_ channels are composed by four ABCC family members’ sulfonylurea receptors (SUR, SUR1, SUR2A or SUR2B) and four pore forming subunits from the inward-rectifier potassium channel 6 (K_ir_6)^20–22^, either the K_ir_6.1 codified by *KCNJ8* or the K_ir_6.2 codified by *KCNJ11*. The subunits forming the K_ATP_ channels are dependent of the cellular type, and SUR1 is mostly present in the β pancreatic cells^20^. This explains why mutations in *ABCC8* have been widely related to type II diabetes mellitus and congenital hyperinsulinism^23–25^. However, how *ABCC8* can cause PAH remains unclear.

In this study, we analyzed a sub-cohort of the Spanish PAH Registry (REHAP) with variants in the *ABCC8* gene. We aimed to characterize the variants with both in silico and in vitro analyses.

## Results

### Description of the cohort

In this study, a total of 744 individuals were analyzed, including 624 patients (579 adults and 45 pediatric) and 120 first degree relatives (from trios and cosegregration studies of positive cases). Baseline characteristics of this cohort are provided in Supplementary Table 1. *ABCC8* variants were identified in 11 patients (1.76% of the patients analyzed). The mean age at diagnosis of these 11 patients was 34 ± 10 years whereas 6 of them were female (54%). Patients 3, 4, 6, 8 and 11 were diagnosed with Idiopathic Pulmonary Arterial Hypertension and patient 4 had a familial form. Patient 7 had PAH associated to Systemic Sclerosis. Patient 10 had a ventricular septal defect, which was repaired at 40 years of age. Patient 2 was clinically diagnosed with possible PVOD associated with systemic sclerosis and HIV infection; PH was diagnosed at 57 years of age. The patient fulfilled all clinical and radiological criteria, so the patient was initially diagnosed with PVOD. DLCO at diagnosis was 22% of predicted value. CT scan showed the typical triad of PVOD: ground glass opacities, interlobular septal thickening and mediastinal lymphadenopathies. The patient also had respiratory insufficiency at rest and significant drop of oxygen desaturation during exercise. Patient 11 was diagnosed at 24 years of age. In the initial diagnosis work-up, restrictive ventricular septal defect was observed. Suprasystemic PAH and right to left shunt were confirmed in the RHC. Considering these findings, ventricular septal defect closure was contraindicated and the patient is currently receiving systemic prostanoids.

Most of the patients showed a Functional Class III (64%) at diagnosis and the mean distance in 6 min’ walk test was 400 ± 120 m. At the moment RHC was performed; Cardiac Index was 2.7 ± 0.7 l/min/m^2^, mean Pulmonary Artery Pressure 58.8 ± 21.8 mmHg, Right Atrium Pressure 7 ± 3 mmHg and Pulmonary Vascular Resistance 10.8 ± 4.7. All the clinical variables are disclosed in Table 1.

**Table 1:** Clinical characteristics of the patients carrying *ABCC8* variants.

Variant	c.211C > T p.(His71Tyr)	c.298G > A p.(Glu100Lys)	c.1429G > A p.(Val477Met)	c.1643C > T p.(Thr548Met)	c.2176G > A p.(Ala726Thr)	c.2422C > A p.(Gln808Lys)	c.2694 + 1G > A	c.3238G > A p.(Val1080Ile)	c.3288_3289del p.(His1097ProfsTer16)	c.3394G > A p.(Asp1132Asn)	c.3976G > A p.(Glu1326Lys)
gnomAD frequency	0	0.00007162	0.000007957	0.00006322	0.0004671	0.00005303	0.000003979	0.00003536	0	0.00003185	0.0001452
PAH type	CHD associated	PVOD	IPAH	IPAH	HPAH	IPAH	CTD associated	IPAH	IPAH	CHD associated	IPAH
Sex	Male	Male	Male	Female	Male	Female	Female	Female	Female	Female	Male
Age of diagnoses, y	24	58	32	27	36	34	26	43	29	NA	31
FC	II	III	III	III	III	III	III	III	II	II	NA
PAP mmHg	150/70/90	47	62/27/39	96/36/58	136/55/91	45/18/29	125/48/78	114/26/55	69/33/45	57/26/38	100/66/77
RAP mmHg	12	2	12	9	4	4	4	9	6	10	6
CI l/min	4.1	5.9	4.8	4.08	4	5.4	5.75	4.6	5.6	2.9	4.8
IC	2.5	3.5	2	2.55	2.15	2.9	3.57	2.7	3.94	1.65	2.3
PVR, WU	18.5	6.7	12	10	19.25	4	12.9	13.2	6.6	8.27	13.2
PRVi, uxm^2^	47.5	11.4	28.68	18.8	35.8	5.9	20.7	17.8	9.3	14.5	29.6
O^2^ Sat % PA	67	68	NA	76	59	98	98	96	NA	74	NA
O^2^ Sat % AO	78	89	93	95	93	NA	68	NA	NA	96	95
NT-pro-BNP	NA	2,180	NA	402	NA	NA	NA	NA	83	327	NA
T6MW m (no O^2^)	484	180	276	525	525	420	463	336	562	366	266
ECO RA cm^2^	28	NA	15	NA	NA	NA	NA	NA	11.9	15	NA
Eco RV mm	64	65	52	39	51	NA	NA	NA	29	51	NA
D_LCO_, %	NA	22.5	92	63	100	72	74	73	92	NA	74
Treatment	PDEi-5, ERAs, systemic prostanoids	PDEi-5, ERAs, inhaled prostanoids	PDEi-5, systemic prostanoids	PDEi-5, ERAs, systemic prostanoids	PDEi-5, ERAs, systemic prostanoids	PDEi-5, ERAs	PDEi-5, ERAs, systemic prostanoids	Systemic Prostanoids	PDEi-5, ERAs	ERAs	PDEi-5, ERAs, selexipag
Survival Time; y	4	4.5	22.5	8	7	12	18	18	9	4	18
Final status	Alive	Exitus	Alive	Alive	Transplant	Alive	Alive	Alive	Alive	Alive	Alive
Variants in PAH genes	*SMAD1* c.27delinsGTAAAG p.(Phe9LeufsTer2)	None	None	None	None	None	None	None	None	None	None
Comorbidities	None	None	None	None	None	None	None	None	None	None	None
Other Commentaries	Restrictive interventricular septal defect and suprasystemic pulmonary hypertension	Clinical diagnosis of PVOD	None	None	None	None	None	None	None	PAH diagnosed after ostium secundum surgery	None

*ExAC* Exome Aggregation Consortium, *PVOD* pulmonary venooclusive disease, CTD connective tissue disease, *CHD* congenital heart disease, *y* years, *FC* functional class, PAP pulmonary arterial pressure, *RAP* right atrial pressure, *CI* cardiac index, *PVR* pulmonary vascular resistance, *PVRi* PVR index, *O*^*2*^* sat* O^2^ saturation, *NT-pro-BNP* B type natriuretic peptide, *T6MW* 6-min walk test, *ECO RA* right atrium echocardiography, *ECO RV* right ventricle echocardiography, *D*_*LCO*_ diffusing capacity, *PDEi-5* phophodiesterase inhibitors, *ERAs* endothelin receptor antagonists, *NA* not available.

### Mutational screening

After filtering for rare variants, 11 heterozygous changes were detected in the *ABCC8* gene, nine of them were missense variants: c.211C > Tp.(His71Tyr), c.298G > A p.(Glu100Lys), c.1429G > A p.(Val477Met), c.1643C > T p.(Thr548Met), c.2176G > A p.(Ala726Thr), c.2422C > A p.(Gln808Lys), c.3238G > A p.(Val1080Ile), c.3394G > A p.(Asp1132Asn) and c.3976G > A p.(Glu1326Lys). An intronic variant: c.2694 + 1G > A; and a frameshift: c.3288_3289del p.(His1097ProfsTer16) were also detected. The patient carrying the *ABCC8* variant c.211C > Tp.(His71Tyr) also carried in heterozygosis a variant in *SMAD1* gene (c.27delinsGTAAAG p.Phe9LeufsTer2). We did not find any Copy Number Variation (CNV) encompassing the genes in the panel (*ACVRL1; GDF2; BMPR1B; BMPR2; CAV1; EIF2AK4; ENG; KCNA5; KCNK3; NOTCH3; SMAD1; SMAD4; SMAD5; SMAD9; TBX4; TOPBP1; SARS2; CPS1; ABCC8; CBLN2; MMACHC*) in this set of patients.

Several of these variants have been described in other pathologies. The variant c.298G > A p.(Glu100Lys) was described in Maturity Onset Diabetes of the Young^25^. Variants c.2176G > A p.(Ala726Thr), c.3976G > A p.(Glu1326Lys) and c.2694 + 1G > A were found in congenital hyperinsulinism patients^26–28^. Lastly, c.2422C > A p.(Gln808Lys) has been described in a diabetes mellitus type 1 patient^27^. For the rest of the variants no relation with any disease has been found, c.211C > Tp.(His71Tyr) and c.3288_3289del p.(His1097ProfsTer16) are described here for the first time. The rs identification of all the variants can be found in Supplementary Table 2.

**Figure 1:**
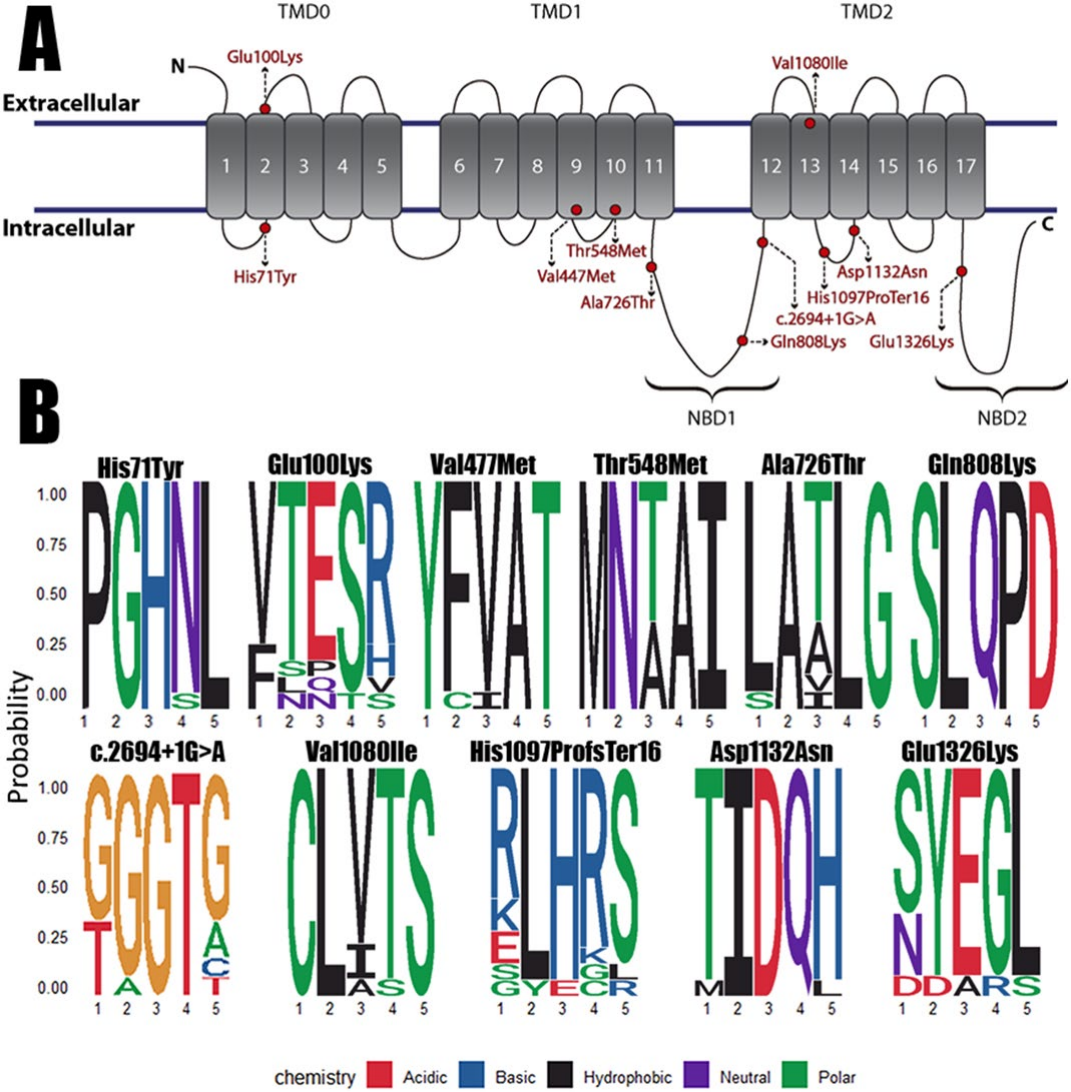
Location of the variants and conservation of the amino acid or nucleotide positions in SUR1. (**A**) Visual scheme of the localization of the variants detected in *ABCC8* after translation to SUR1 in our cohort (red). Five variants are located in the cytoplasmic domain, three in the transmembrane domain and one in the extracellular domain. The variants are marked as a yellow circle; the positions have been estimated from Uniprot SUR1 entry (#Q09428). (**B**) Sequence logos from the comparison of 14 *ABCC8* reference sequences from different animals to evaluate the conservation of the mutated positions. The variants are located in the 3rd position of the logo. The nature of the amino acid nature is also stated in a legend with the exception of c.2694 + 1G > A.

Most of the variants are located in cytoplasmic domains: c.211C > T p.(His71Tyr), c.2176G > A p.(Ala726Thr), c.2422C > A p.(Gln808Lys), c.3288_3289del p.(His1097ProfsTer16), c.3394G > A p.(Asp1132Asn) and c.3976G > A p.(Glu1326Lys). Three are located in transmembrane domains: c.1429G > A p.(Val477Met), c.1643C > T p.(Thr548Met) and c.3238G > A p.(Val1080Ile). Only one is located in an extracellular domain (Fig. 1); c.298G > A p.(Glu100Lys).

Interestingly, six of these variants are present in functional SUR1 positions. Two variants; c.211C > T p.(His71Tyr) and c.298G > A p.(Glu100Lys); are located at the first transmembrane domain (TMD0), a region that interacts closely with K_ir_6.2. While the resulting four variants are located within Nucleotide-Binding Domains (NBD) where ATP binds, concretely, c.2176G > A p.(Ala726Thr), c.2422C > A p.(Gln808Lys) and c.2694 + 1G > A (after exon 22) at NBD1; and c.3976G > A p.(Glu1326Lys) at NBD2.

Only two families decided to undertake cascade testing. Patient 4 (carrying p.Thr548Met) inherited the variant from his mother (unaffected carrier), his father was WT. In the case of patient 7 (carrying c.2694 + 1G > A), her mother was WT and her father was deceased years before the enrollment in the registry, so the variant was either de novo or inherited from her father.

### Variant in silico analysis and classification

The ACMG prediction score was used to determine the grade of evidence to support the pathogenicity (PP3) and benignity (BP4) of the variants: BP4 (0–2), Variant of Uncertain Significance (VUS) (2–4) and PP3 (4–7). In the case of frameshift or exon skipping mutations (PVS1) was stated. A summary of all the predictions is detailed in Tables 2 and 3. Variants c.298G > A p.(Glu100Lys), c.2176G > A p.(Ala726Thr) and c.3238G > A p.(Val1080Ile) were classified as VUS. c.211C > Tp.(His71Tyr), c.1429G > A p.(Val477Met), c.1643C > T p.(Thr548Met), c.2422C > A p.(Gln808Lys), c.2694 + 1G > A, c.3976G > A p.(Glu1326Lys) and the *SMAD1* variant c.27delinsGTAAAG p.(Phe9LeufsTer2) were classified as likely pathogenic. Lastly, c.3288_3289del p.(His1097ProfsTer16) and c.3394G > A p.(Asp1132Asn) were classified as pathogenic.

**Table 2:** In silico analysis to predict the effect of the variants identified in the ABCC8 gene.

cDNA and protein position	Annovar impact	CADD	Sift	Polyphen2	MutAssesor	Fathmm	VEST	Score	ACMG Classification
c.211C > T p.(His71Tyr):	–	Damaging	Damaging	Probably damaging	Damaging	Damaging	Damaging	5.5/7	Likely pathogenic
c.298G > A p.(Glu100Lys)	MODERATE	Possibly damaging	Benign	Benign	Benign	Damaging	Possibly damaging	2.5/7	VUS
c.1429G > A p.(Val477Met)	MODERATE	Damaging	Damaging	Damaging	Possibly damaging	Damaging	Damaging	6/7	Likely pathogenic
c.1643C > T p.(Thr548Met)	MODERATE	Damaging	Damaging	Damaging	Possibly damaging	Damaging	Possibly damaging	5.5/7	Likely pathogenic
c.2176G > A p.(Ala726Thr)	MODERATE	Damaging	Benign	Benign	Benign	Damaging	Benign	2.5/7	VUS
c.2422C > A p.(Gln808Lys)	MODERATE	Damaging	Possibly damaging	Probably damaging	Benign	Damaging	Damaging	4.5/7	Likely pathogenic
c.2694 + 1G > A	–	Damaging	–	–	–	–	–	1/7	Likely pathogenic
c.3238G > A p.(Val1080Ile)	MODERATE	Damaging	Benign	Benign	Benign	Damaging	Benign	2.5/7	VUS
c.3288_3289del p.(His1097ProfsTer16)	HIGH	–	–	–	–	–	–	1/7	Pathogenic
c.3394G > A p.(Asp1132Asn)	MODERATE	Damaging	Damaging	Damaging	Damaging	Damaging	Possibly damaging	6/7	Pathogenic
c.3976G > A p.(Glu1326Lys)	MODERATE	Damaging	Possibly damaging	Benign	Benign	Damaging	Damaging	4/7	Likely pathogenic
*SMAD1* c.27delinsGTAAAG p.(Phe9LeufsTer2)	–	–	–	–	–	–	–	–	Likely pathogenic

CADD ranges: s > 14 damaging, s in [11,14) possibly damaging, SIFT ranges: benign s > 0.23, possibly damaging s in (0.06, 0.23), damaging s ≤ 0.06. Polyphen2 ranges: benign s < 0.03, possibly damaging s in (0.03, 0.3), damaging s > 0.3. MutAssesor ranges: benign s < 1.12, possibly damaging s in [1.12, 1.8), damaging s > 1.8. Vest ranges: Benign s < 0.17, possibly damaging s in [0.17, 0.65), damaging s ≥ 0.65.

**Table 3:** Splicing in silico analysis of the variants detected in the *ABCC8* gene.

cDNA and protein position	ada.Pred	rf.Pred	NetGene2	NNSPLICE	HSF	Minigene assay
c.211C > T p.(His71Tyr)	–	–	Neutral	Neutral	Activation of an exonic cryptic donor site. Alteration of an exonic ESE site. Potential alteration of splicing	Neutral
c.298G > A p.(Glu100Lys)	–	–	Neutral	Neutral	Alteration of an exonic ESE site. Potential alteration of splicing	Neutral
c.1429G > A p.(Val477Met)	–	–	Neutral	Splice site score reduced	Creation of an exonic ESS site. Alteration of an exonic ESE site. Potential alteration of splicing	Neutral
c.1643C > T p.(Thr548Met)	–	–	Neutral	Neutral	Neutral	N.A
c.2176G > A p.(Ala726Thr)	–	–	Neutral	Neutral	Neutral	–
c.2422C > A p.(Gln808Lys)	–	–	Creation of a novel splicing acceptor site	Creation of a novel splice site	Creation of an exonic ESS site. Alteration of an exonic ESE site. Potential alteration of splicing	N.A
c.2694 + 1G > A	Possibly damaging splicing site | s > 0.612	Possibly damaging splicing site | s > 0.598	Donor site affected (0.0 confidence)	Neutral	Creation of an exonic ESS site. Potential alteration of splicing	N.A
c.3238G > A p.(Val1080Ile)	–	–	Acceptor splice sites scores increase	Neutral	Neutral	–
c.3288_3289del p.(His1097ProfsTer16)	–	–	Creation of a novel splicing acceptor site. Splice acceptor site score increases	Neutral	Creation of an exonic ESS site. Alteration of an exonic ESE site. Potential alteration of splicing	N.A
c.3394G > A p.(Asp1132Asn)	–	–	Neutral	Splice site score reduced	Alteration of an exonic ESE site. Potential alteration of splicing	Exon skipping
c.3976G > A p.(Glu1326Lys)	–	–	Creation of a novel splicing acceptor site	Neutral	Creation of an exonic ESS site. Potential alteration of splicing	N.A

The splicing effect predictions were taken into account to decide whether or not experimental confirmations needs to be performed, scores are detailed in Table 3. The variants where 2 predictors showed alterations were automatically considered candidates. Exceptions were made for c.298G > A p.(Glu100Lys) due to its location within an intron–exon junction, c.211C > T p.(His71Tyr) because of the possible partial loss of the exon, and c.1643C > T p.(Thr548Met) that was chosen to assess a missense “control” variant in the assay.

Overall, most of the variants were conserved among the evolution, c.211C > T p.(His71Tyr), c.2422C > A p.(Gln808Lys), c.2694 + 1G > A and c.3394G > A p.(Asp1132Asn were highly conserved. Whereas c.1429G > A p.(Val477Met), c.2422C > A p.(Gln808Lys) and c.3976G > A p.(Glu1326Lys) were conserved mostly in vertebrates so there is some variability when moving farther from humans. Lastly, c.298G > A p.(Glu100Lys), c.1643C > T p.(Thr548Met), c.2176G > A p.(Ala726Thr) and c.3238G > A p.(Val1080Ile) were not conserved (Fig. 1B).

### Minigene assay

**Figure 2:**
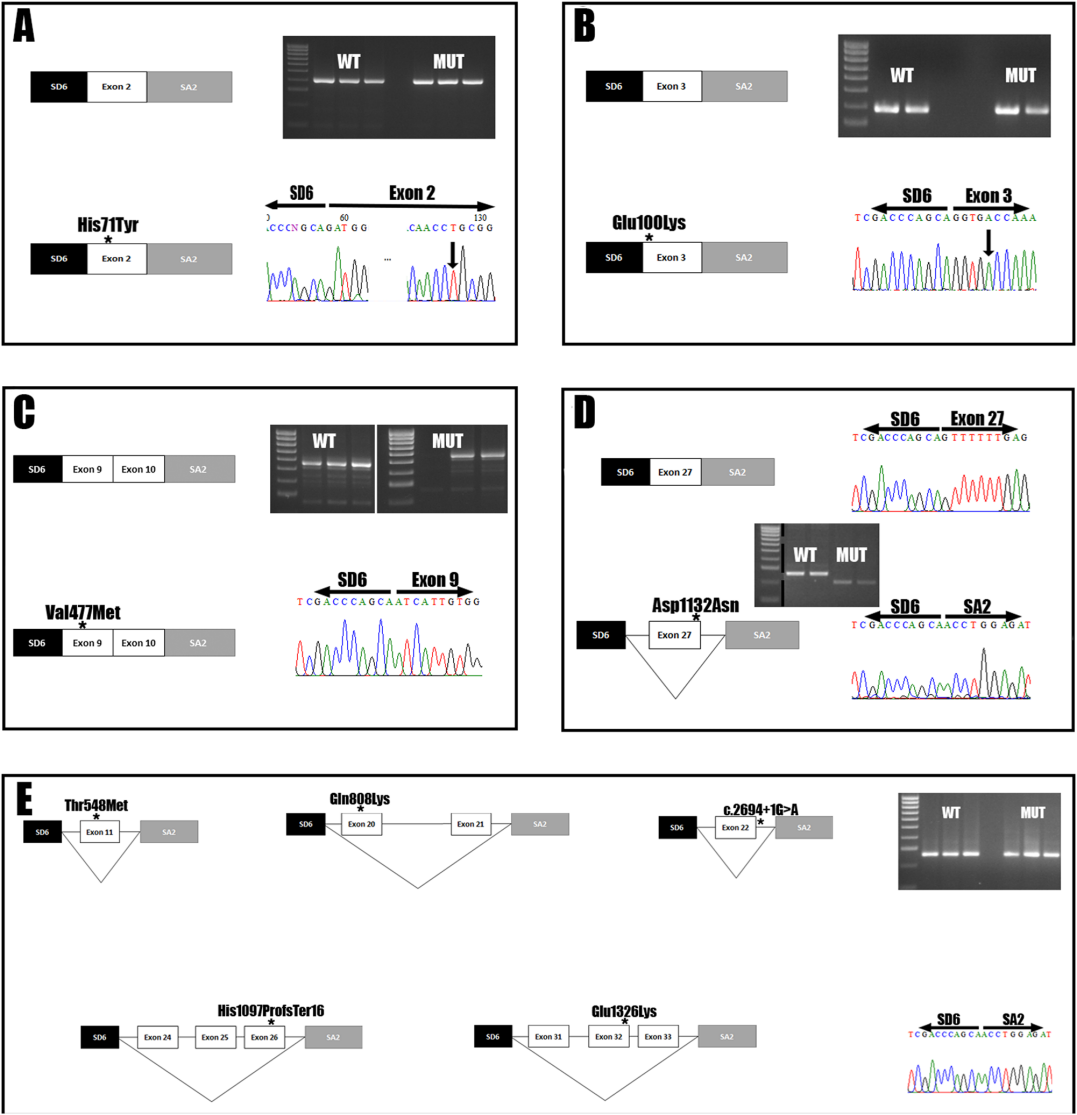
Minigene assay highlight the pathogenicity of c.3976G > A and a complex regulation in *ABCC8* transcription. Every subset of images includes a box scheme of the mRNA analysis results, its representing gel electrophoresis and Sanger sequencing results from the exon junction found. (**A**) The p.His71Tyr variant located in exon 2 does not alter splicing. (**B**) The p.Glu100Lys variant located in the exon 3 does not alter splicing. (**C**) The p.Val477Met variant located in exon 9 does not impair splicing; both exon 9 and exon 10 are detectable. (**D**) The p.Asp1132Asn variant located in exon 27 induces an exon skipping event. The gel electrophoresis image is cut because two different constructs were tested in the same gel. (**E**) Exons 11, 20, 21, 22, 24, 25, 26, 31, 32 and 33 were not transcribed in both wild type and variant constructs making it impossible to correctly test the splicing effects of the described variants. Unedited images for all the gels are provided as supplementary material.

Based on the in silico analysis we selected 9 out of 11 variants for the minigene assay: c.211C > T p.(His71Tyr), c.298G > A p.(Glu100Lys), c.1429G > A p.(Val477Met), c.1643C > T p.(Thr548Met), c.2422C > A p.(Gln808Lys), c.2694 + 1G > A, c.3288_3289del p.(His1097ProfsTer16), c.3394G > A p.(Asp1132Asn), c.3976G > A p.(Glu1326Lys). Only four out of nine constructs worked as expected (c.211C > T p.(His71Tyr), c.298G > A(p.Glu100Lys), c.1429G > A p.(Val477Met) and c.3394G > A p.(Asp1132Asn); Fig. 2A–D) while the constructs encoding the variants located in/or near the exons 20, 21, 22, 24, 25, 26, 31, 32 and 33 did not transcribe any of the *ABCC8* exons present in the construction (Fig. 2E). The variants c.211C > T p.(His71Tyr), c.298G > A p.(Glu100Lys) and c.1429G > A p.(Val477Met) did not alter the correct splicing of the exons 2, 3, 9 and 10, as they were detectable in both the wild type and the mutated construct (Fig. 2A–C). However, c.3394G > A p.(Asp1132Asn) induced the complete skipping of the exon 27 due to the alteration of an exonic splicing enhancer site (Fig. 2D), this change causes a frameshift leading to the creation of a stop codon in the former exon 28, producing a truncated protein of 1,118 amino acids. All the constructs with coding exons that were not detected in the assay (neither in the mutated nor wild type) were sequenced to confirm the integrity of the inserts.

### *ABCC8* regulation analysis

The *ABCC8* gene introns have a high amount of regulatory regions, enhancing positions, LINEs and SINEs (Supplementary Fig. 1). Also, there are four enhancer regions identified in the GENEHANCER study^29^ that are located in regions surrounding the exons that did not transcribe in our minigene constructs: GHJ017432 surrounding exon 11; GH11J017412 including exons 18, 19 and 20; GH11J017411 before exon 22; GH11J017404 comprehending exons 26, 27 and 28; lastly, GH11J017401 upwards of exon 30. These enhancing regions are used by a wide variety of transcription factors detailed in Supplementary Table 3. We also used the MaxEntScan tool from *Human Splicing Finder* to measure the strength of the splice sites encoding the exons used in the minigenes. We did not find a correlation between the predicted strength of the site and the expression of the minigene construct (Supplementary Table 4).

### Protein modeling

**Figure 3:**
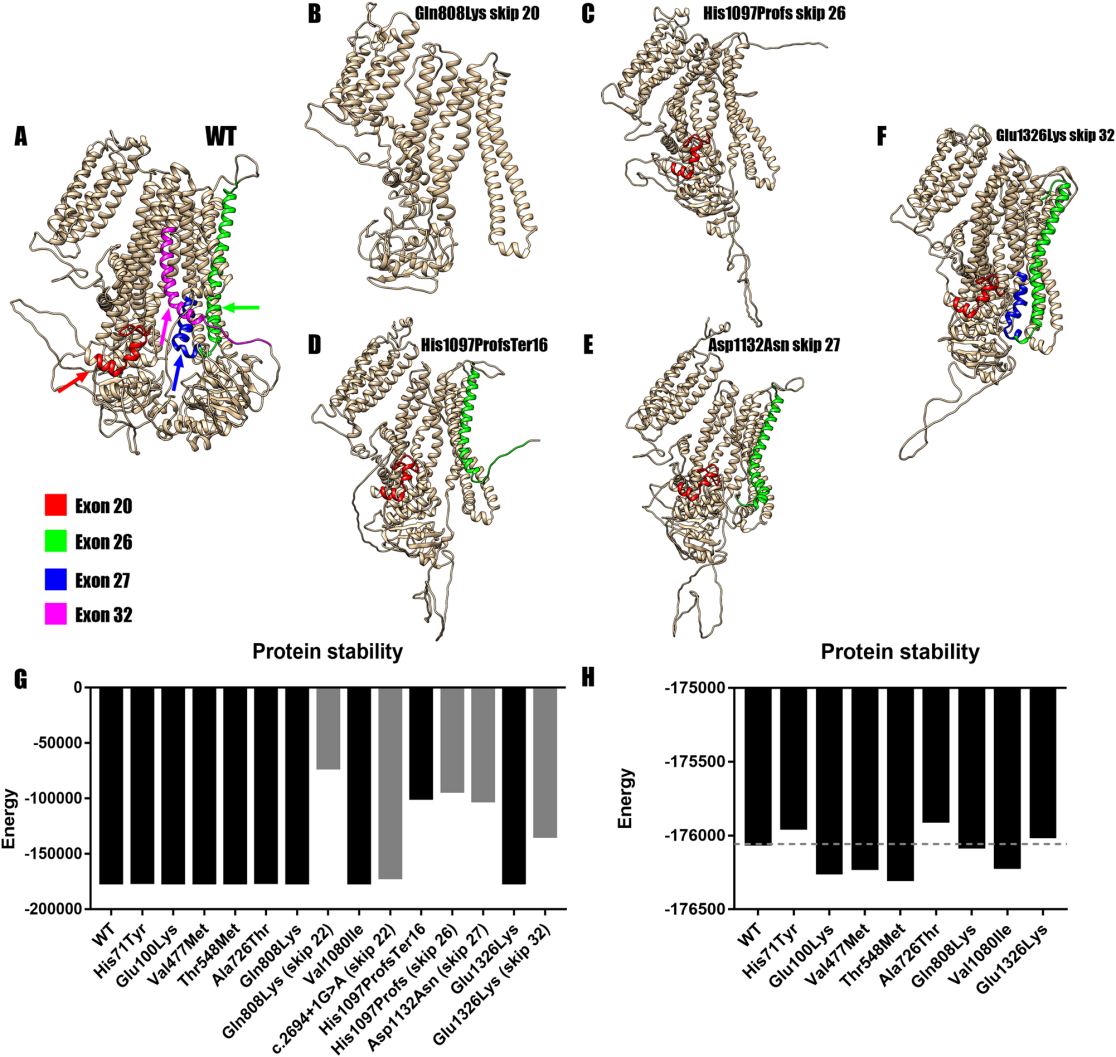
Protein modeling of the most unstable variants. (**A**) Wild type SUR1 marked for the target exon. Red marks exon 20, green for exon 26, blue for the exon 27 and magenta for exon 32. (**B**) Structure for Gln808Lys in the case of an exon skipping event. The resulting protein loses 747 amino acids and so its structure is altered. (**C**) Structure of His1097ProfsTer16 skipping exon 26. The protein loses 527 amino acids altering its structure. (**D**) Structure of the resulting protein of His1097ProfsTer16. The protein loses a total of 471 amino acids and its structure is altered. (**E**) Structure after the mRNA confirmed skipping of exon 27 due to p.(Asp1132Asn). The skipping of exon 27 truncates the protein making it lose 463 amino acids and altering its structure. (**F**) Structure for Glu1326Lys after the skipping of exon 32. The protein loses 290 amino acids; the structure is altered but not as much as in earlier skips. (**G**) Comparison of DOPE energies between the models of the wild type (WT) SUR1 and the mutated proteins (Black for missense models and gray for exon skippings). All the amino acid substitutions showed similar stability when compared to the wild type. However, Gln808Lys (exon 22), His1097ProfsTer16, His1097ProfsTer16 (skip 26), Asp1132Asn (skip 27) and Glu1326Lys (skip 32) showed a much lower stability which would mean more unstable proteins. (**H**) Detailed graphic comparing all the missense variants and the WT template. None of the variants showed high DOPE energy variation when compared with the WT.

All variants that were not experimentally validated, c.1429G > A p.(Val477Met), c.2176G > A p.(Ala726Thr), c.2422C > A p.(Gln808Lys), c.3238G > A p.(Val1080Ile), c.3976G > A p.(Glu1326Lys) did not alter the protein structure nor its stability (Fig. 3G,H) based on an in silico analysis when compared with the wild type (Fig. 3A). The simulated skipping of exon 20 in c.2422C > A p.(Gln808Lys) was predicted to be the most destabilizing both at structure (Fig. 3B) and stability level (Fig. 3G), producing a protein of 836 amino acids. However, the skipping of exon 22 in c.2694 + 1G > A barely affected its stability (Fig. 3H), probably due to the predicted in frame loss of only 46 amino acids. For c.3288_3289del p.(His1097ProfsTer16), both the frameshift and the simulated skipping of exon 26 yielded a truncated protein of 1,111 and 1,056 amino acids respectively, that showed altered structure (Fig. 3C,D) and protein stability (Fig. 3G), so this variant can be considered equally pathogenic whether it affects splicing or not. The simulated skipping of exon 32 in c.3976G > A p.(Glu1326Lys) produces a protein of 1,292 amino acids that alters slightly its structure (Fig. 3F) and protein stability (Fig. 3H). Regarding the minigene validated variants, only the c.3394G > A p.(Asn1132Asp) caused major alterations in protein structure (Fig. 3E) and its stability (Fig. 3G). All the missense variants, both confirmed and unconfirmed, were compared in detail (Fig. 3H). Concerning the stability, the variations accounted were of less than ± 500 between them and the wild type. We then analyzed all the missense variants with Missense3D, which showed no structural alterations caused by the amino acid substitutions.

All the homology models generated by Phyre2 showed less stability than the template used for its modeling (Supplementary Fig. 2), as expected when comparing an experimentally obtained template and simulated (modeled) data.

## Discussion

PAH is a devastating disease if it is not treated. During the last decade the application of high throughput sequencing has increased the knowledge on the genetic basis of PAH, becoming a strong tool with high potential to find out novel treatments^30^.

In this study we identified by a custom NGS targeted sequencing panel of 21 genes (HAP v1.2), eleven variants in *ABCC8* gene after screening 624 patients from the Spanish PAH Registry (REHAP).

This set of patients with *ABCC8* variants compose a 1.76% of our cohort and show a younger age of diagnosis when compared with other recent studies^9,31^. Also, they seem to show more severe forms of the disease with higher PAP values and FC, but this may be due to the small number of patients in our cohort referring variants in this gene. Regarding the etiologies, this set is composed mostly by IPAH patients, with two patients of CHD-APAH and single patients of HPAH, CTD-APAH and, for the first time, an associated form of PVOD.

None of them show any clinical feature related to congenital hyperinsulism as mutations in the *ABCC8* gene have been described in this disorder^23–25^.

Most of the detected *ABCC8* variants were located in regions within or near exon–intron boundaries so the possible effects in the splicing had to be taken into account. After using pathogenicity and splicing predictors, we tested nine variants through minigene assay to determine the effect in splicing at mRNA level. Only four of the constructs worked as expected, confirming the correct processing of c.211C > T p. (His71Tyr), c.298G > A p.(Glu100Lys) and c.1643C > T p.(Thr548Met) located in exons 2, 3 and 9, respectively. The variant c.3394G > A p.(Asp1132Asn) causes an exon skipping of exon 27 of *ABCC8*, so we propose to change its nomenclature at RNA level from r.3394G > A to r.del3330_3399 and at protein level from p.(Asp1132Asn) to p.?. Unfortunately, constructs did not work in the five remaining variants; therefore, the possible splicing effect for these variants remains unknown. This may be due to neither the wild type nor the mutated exons were transcribed in our constructs. As a first approach, we tried to perform the minigene assay using standard length minigene constructs. With a fragment containing the exon of interest and, at least, 100–200 bp around 3′ and 5′, this approach allowed us to detect the exon 27 skipping, but none of the other constructs transcribed the exons, so we decided to use longer constructs with 800–1,500 bp around including multiple exons. With this approach we were able to confirm that c.211C > T p.(His71Tyr), c.298G > A p.(Glu100Lys) and c.1643C > T p.(Thr548Met) do not alter the splicing. Thus, we hypothesize that this inability to transcribe those exons may have been mediated by the absence of regulatory elements for these exons. HeLa cells express *ABCC8* (Protein Atlas) and should have all the cellular machinery needed to do it, so we discarded a tissue specificity problem. The UCSC genome browser analysis of *ABCC8* revealed regulatory regions before or within the exons 11, 18, 19 20, 26, 27, 28, 29 and 30 (Supplementary Fig. 1). One of them has binding sites for up to 40 transcription factors. Length of this regions are variable, ranging from less than 200 up to 2,000 bp, and strikingly, the constructs that worked (exons 2, 3, 9 and 10) did not have any regulatory element nearby, but exon 27 was right in the middle of one, and apparently 351 bp of it (including the exon) were enough to allow it to be transcribed.

Furthermore, in the non-working constructs we had most of the regulatory region within exon 11 (GH11J017432); in the case of exon 20 and 21, the 1,200 bp insert included 800 bp of the regulatory region encoding exon 20 (GH11J017412); for exon 22, we missed the region GH11J017411 by 250 bp, this region is only 151 bp long; in the case of the construct 24–25–26 we had 500 bp of the GH11J017404 which encodes the end exon 25 and the whole exon 26; and lastly, the construct with exons 31–32–33 had its closest regulatory region 3,000 bp upstream of exon 31. The regulation by upstream regions and the need to use clusters of exons is not something new^32^, but it arises the limitations of testing splicing variants in vitro using minigenes instead of specific tissues, the larger the gene the more complicated its regulation will be as we can see in *ABCC8*. Thus, we would need to access specific tissue in order to properly test the pathogenicity of the resulting splicing variants.

In order to facilitate the in silico analysis, due to the unavailability of patient’s specific tissue, we chose a bioinformatics approach using protein homology modeling and protein stability analyses. We modeled the effect of the missense changes and the confirmed skipping variants. These results highlight how exon skipping events in most of the situations would yield an unstable protein that may be degraded, unable to fold correctly and probably non-functional.

All the amino acid changes simulated did not affect the overall structure and stability of the protein, but some of them are located in functional positions. p.(His71Tyr) and p.(Glu100Lys) are located in the region that interacts closely with the gating of the K_ir_6.2 pore domain^33^. It is likely that additional changes affecting NBD1 (p.Ala725Thr, p.Gln808Lys) and NBD2 (p.Glu1326Lys) alter the catalytic activity of SUR1, and therefore, its role to provide stability and regulate K_ir_6.2 gating. The variants located at the transmembrane domains could not have a high functional effect, but electrophysiology studies are needed.

It is noteworthy that the patient with the p.(His71Tyr) variant was also carrying in heterezygosity an indel in the *SMAD1* gene (c.27delinsGTAAAG), producing an early frameshift in *SMAD1* p.(Phe9LeufsTer2) that yields the complete loss of the allele. Mutations in *SMAD1* have been related to PAH, and functional studies have confirmed the decrease in signaling due to missense variants^34^, but the exact molecular mechanism that leads to PAH remains unknown^35^. Thus, we propose that PAH in this patient may be caused by the combination of both variants, where one of them could act as a 2nd hit needed to start PAH pathogenesis.

Bohnen et al.^9^ have already shown that amino acid substitutions affect the correct electrophysiological function of the SUR1-K_ir_6.2 complex. Their study is performed using cDNA constructs encoding the variants to test. This means that all the variants analyzed will be treated as missense, and one would be losing all the effects from the possible alterations of the splicing process. Also, mRNA processing can play a vital role in the amount of mutant protein that is traduced; the mutated alleles can be degraded reducing the theoretical 50–50%.

Furthermore, the assembly of the SUR1-K_ir_6.2 complex should be random, yielding channels with 1, 2, 3 or 4 SUR1 wild type or mutated proteins (if not degraded) making almost compulsory to carry out several measurements of activity by single channel patch clamp to be able to appreciate the real effect. Using cell culture to validate novel PAH related genes is useful, but animal models are still needed to fully associate loss of function in *ABCC8* to PAH. Until now, none of the mice models referring *ABCC8* mutations or its complete gene knockout have been described to develop PAH according to Mouse Genetic Information data, weakening the link between the gene and the disease. *ABCC8* expression pattern does not exactly fit with PAH known pathogenesis, as we would expect SUR1 to be highly expressed in cardiac and smooth muscle cells, but this is not the case, those tissues express much more the closely related SUR2 in combination with both K_ir_6.1 and K_ir_6.2^36^. SUR1 is mainly expressed in pancreas, where its link to congenital hyperinsulinism^37^ and neonatal diabetes^38^ has been proven many years ago.

However, SUR1 is detectable in lung, proximal pulmonary arteries^9^ and heart atrium^39^. Therefore, the only way to link the loss of function in *ABCC8* with PAH is if it induces a vasoconstrictor effect similar to hypoxia. In hypoxia, K_ATP_ channels are inhibited in the pulmonary smooth muscle cells, inducing Ca^2+^ uptake and thus, an increase in contraction and proliferation that could start PH^18^. This would end up increasing shear stress, inducing endothelial cells to proliferate^40,41^.

In conclusion, we report eleven novel changes in *ABCC8* gene found in PAH patients. Thanks to in vitro biochemical analyses and in silico tools we were able to classify them according to ACMG: two as pathogenic, six as likely pathogenic and three as VUS (Table 4).

**Table 4:** Summary table with the conclusions for each *ABCC8* variant.

cDNA and protein position	Conclusion
c.211C > T p.(His71Tyr):	Likely pathogenic, located in a gating regulatory region, confirmed missense, predictors agree
c.298G > A p.(Glu100Lys)	VUS, located in a gating regulatory region, confirmed missense, predictors do not agree
c.1429G > A p.(Val477Met)	Likely pathogenic, confirmed missense, all the predictors agree
c.1643C > T p.(Thr548Met)	Likely pathogenic, all the predictors agree, inconclusive minigenes, predicted non splicing altering
c.2176G > A p.(Ala726Thr)	VUS, located in NBD1, predictors do not agree, predicted non splicing altering
c.2422C > A p.(Gln808Lys)	Likely pathogenic, located in NBD1, inconclusive minigenes, possibly splicing altering
c.2694 + 1G > A	Likely pathogenic, located in NBD1, inconclusive minigenes, possibly splicing altering
c.3238G > A p.(Val1080Ile)	VUS, unconfirmed missense, predictors do not agree, predicted non splicing altering
c.3288_3289del p.(His1097ProfsTer16)	Pathogenic, clearly dysfunctional protein
c.3394G > A p.(Asp1132Asn)	Pathogenic, minigenes confirmed exon skipping, induces a frameshift
c.3976G > A p.(Glu1326Lys)	Likely pathogenic, located in NBD2, inconclusive minigenes, possibly splicing altering

## Materials and methods

### Description of the cohort

Since November 2011, genetic studies have been offered to all patients included in the Spanish Registry of Pulmonary Arterial Hypertension (REHAP) with idiopathic and hereditable forms of PAH, and PVOD^2,30^. All the methods were performed in accordance with the ethical principles of the European Board of Medical Genetics and the 2015 ERS/ESC guidelines for the diagnosis and treatment of pulmonary hypertension to provide accurate information on the range of options available to make informed decisions and allow equal access to genetic counseling and testing^1^. Pre and post-test genetic counseling was provided. All patients or legal tutors included in the analysis gave their informed consent. The project was approved by the ethical committee for scientific research of the Hospital Universitario 12 de Octubre (*Comité Ético de Investigación Clínica—Hospital 12 de Octubre).*

First degree relatives to affected probands were clinically screened when available. Cascade or cosegregration genetic tests were also offered. When an unaffected carrier was identified, a complete diagnostic work-up was performed, including electrocardiogram, echocardiogram, N-terminal pro-brain natriuretic peptide (NT-proBNP) and 6 min’ Walk Test. Due to the typical phenotype, DLCO was also determined in healthy carriers. This evaluation is periodically repeated. When a sustained suspicion of early stage PAH was observed, right heart catheterization (RHC) was performed to rule out the condition.

### Targeted panel sequencing

We analyzed all the patients (744) using a custom panel (HAP v1.2) designed with NimbleDesign (Roche, Basel, Switzerland), which includes a total of 21 genes (disease associated and research genes): *ACVRL1; GDF2; BMPR1B; BMPR2; CAV1; EIF2AK4; ENG; KCNA5; KCNK3; NOTCH3; SMAD1; SMAD4; SMAD5; SMAD9; TBX4; TOPBP1; SARS2; CPS1; ABCC8; CBLN2; MMACHC*. Next, fragmentation and capture of the target regions were performed with SeqCap EZ Choice Enrichment Kit (Roche, Basel, Switzerland) and sequencing was carried out in Illumina MiSeq platform (Illumina, San Diego, USA). We developed an in-house bioinformatics pipeline to analyze the raw data. After the filtering of the relevant variants, we validated the candidate variants through traditional Sanger Sequencing. Finally, we performed the review, classification and interpretation of the variants according to the American College of Medical Genetics and Genomics (ACMG) guidelines^42^. We performed a CNV analysis applying the custom framework *LACONv* (https://github.com/kibanez/LACONv), which have been developed in-house. It detects gains and losses in genes included in the HAP v1.2 panel. The minimal depth by sample to be considered was 20×. The minimal depth in genomic intervals to be considered was 15×. The doses rate threshold for deletions is 0.60. The doses rate threshold for duplications was 1.20. The Z score threshold to establish CNVs (deletions) was − 2.000.

### Sanger sequencing

A standard PCR was carried out using GoTaq Green master mix (Promega, Madison, USA) with annealing temperatures ranging between 58–65 °C depending on the construct (Supplementary Teble 2). PCR products were purified using the ExoSAP-IT kit (Thermo Fisher, Waltham, USA). When we had to purify bands from gel electrophoresis, the PCR cleanup gel extraction kit (Macherey–Nagel, Düren, Germany) was used. The sequencing was performed using the BigDye terminator v3.1 Cycle Sequencing kit (Thermo Fisher, Waltham, USA). We then precipitated the sequencing products and analyzed them in an ABI PRISM 3100 genetic analyzer (Thermo Fisher, Waltham, USA). Finally, sequences were aligned to the corresponding reference sequence from ENSEMBL (ENST00000389817.7).

### Variant in silico analyses

The *ABCC8* variants were analyzed to predict mutational effect with the following software: dbNSFP^43^, Annovar^44^, CADD^45^, Sift^46^, Polyphen2^47^, MutAssesor^48,49^, Fathmm^50^ and VEST^51^. Regarding of the effect assessed by the predictors, we assigned each variant a prediction score with a maximum of 7 points following this criteria: 1 = “Damaging/High”, 0.5 = “Possibly damaging/Moderate” and 0 = “Benign”, the score was used to sum up the level of evidence from the predictors in order to classify the variants following the ACMG guidelines. Because most of the variants were located near or within exon–intron junctions, the following splice effect predictors were also used: Alamut Interactive Software (Interactive Biosoftware, Rouen, France), NetGene2^52^, NNSplice^53^ and Human Splicing Finder^54^. We then compared splicing prediction data with our minigene assay results in the cases we could. The conservation of amino acids or nucleotides was represented as sequence logos using data from the *ABCC8* Ensembl reference sequences of 14 different species (human, chimp, mouse, rat, cat, dolphin, cow, elephant, alpaca, platypus, chicken, zebrafish, coelacanth and lamprey) with the R package ggseqlogos^55^.

### Minigene design

For the minigene assay we followed two different approaches. First, we amplified fragments containing the exon of interest and at least 80 bp of each flanking intronic regions. Due to the inconclusive results yielded by the standard constructs (lack of *ABCC8* exons transcription in both wild type and mutated states), we increased the length of the fragments to a range between 1–1.5 kb, including multiple exons in the same construct. All the primers used for the PCR amplification are available in Supplementary Table 2. The PCR was carried out using Phusion Hot Start high fidelity polymerase (Thermo Fisher, Waltham, USA), and the cycling conditions are as follows: 98 °C for 3 min, 35 cycles of 98 °C for 10 s, each primer pair melting temperature for 45 s, 72 °C for 30 s and a last annealing step at 72 °C for 5 min. After checking the amplification with an agarose gel electrophoresis, we digested the PCR products using either XhoI/EcoRI/BamHI and NheI (NZYtech, Caparica, Portugal), so they could be subcloned into the pSPL3 vector with a T4 DNA ligase (Canvax, Córdoba, Spain) at 22 °C for 15 min using a 3:1 insert:vector ratio. Finally, we transformed NZYStar competent cells (NZYtech, Caparica, Portugal) and confirmed the cloning by PCR using each insert specific primers.

### Site directed mutagenesis

The mutated constructs for each of the variants were generated using the NZYmut site directed mutagenesis kit (NZYtech, Caparica, Portugal). We carried out the reaction using 100 ng of the wild type plasmid following manufacturer’s protocol. All the primer sequences used for the reactions are indicated in Supplementary Table 2. For the fragments encoding c.1643C > T p.(Thr548Met), c.2694 + 1G > A and c.3394G > A p.(Asp1132Asn) the mutated inserts were synthesized by IDT (Coralville, Iowa, USA).

### Cell culture and transfection

HeLa cells (ATCC, Manassas, USA) were cultured in DMEM (Thermo Fisher, Waltham, USA) supplemented with 10% Fetal Bovine Serum (FBS) (Thermo Fisher, Waltham, USA), 1% l-glutamine/streptomycin/penicillin (Lonza, Basel, Switzerland), at 37 °C with 5% CO_2_ and a humidified atmosphere. We seeded approximately 300,000 cells per well into a 6 well plate, when 80–90% confluence was achieved, we carried out the transfection using 2.5 µg of plasmid DNA and Lipofectamine 2000 (Thermo Fisher, Waltham, USA) in a 1:3 reagent: DNA ratio per well following manufaturer’s recommendation. The wild type and mutated constructs were transfected by triplicate.

### Minigene assay

We harvested the cells 36 h post-transfection, RNA was extracted using the Nucleospin RNA (Macherey–Nagel, Düren, Germany) following manufacturer’s protocol and a RT-PCR was carried out using 1 µg of RNA with the First Strand cDNA Synthesis kit (Canvax, Córdoba, Spain). Then, we used 2 µL of cDNA as template for a PCR using the Phusion Green Hot Start II High Fidelity polymerase (Thermo Fisher, Waltham, USA) with the SD6 5′-TCTGAGTCACCTGGACAACC-3′and SA2 5′-ATCTCAGTGGTATTTGTGAGC-3′ primers that anneal in the pSPL3 vector exons. The amplification was performed as follows: 98 °C for 3 min, 35 cycles of 98 °C for 10 s, 58 °C for 30 s and 72 °C for 30 s, finally, 72 °C for 7 min. PCR products were separated by electrophoresis in a 2% agarose gel to analyze changes in pSPL3 transcript pattern, to confirm splicing alterations, we sequenced the bands using BigDye Terminator v3.1 Cycle Sequencing Kit (Thermo Fisher, Waltham, USA). All the working constructs have been deposited in Addgene (Watertown, USA) pSPL3-ABCC8-2 (#135916), pSPL3-ABCC8-3 (#135917), pSPL3-ABCC8-9-10 (#135918) and pSPL3-ABCC8-27 (#135919).

### *ABCC8* regulation analysis

We evaluated the gene regulation network and binding motifs sequences using the UCSC genome browser^56^.

### Protein modeling and protein stability analyses

We generated homology models for the different SUR1 variants with Phyre2^57^. The models were then superposed with the wild type SUR1 in Chimera^58^. For the protein stability analysis, the models were built considering the protein structure C63O downloaded from the Protein Data Bank^22^ as a template (indicated by Phyre2). Next, we obtained the discrete optimized protein energy (DOPE), which provides a measure of protein folding stability^59^ and that is implemented in MODELLER^60^. When the predictors could not rule out possible splicing alterations, we simulated the effect of a whole exon skipping in the sequence (and the amino acid substitution or frameshift in the protein structure). The effects in the protein sequence were predicted by editing the reference sequence in Snapgene viewer (GSL Biotech, Chicago, USA).

For the missense variants, we used the Missense3D server^61^ to evaluate changes in protein conformation caused by amino acid substitution events.

## Supplementary information

Supplementary Information.

## Data Availability

Data supporting the findings of this study are available on request from the corresponding author. The data is not publicly available due to privacy or ethical restrictions.
