# Supplementary material for: Characterization of rare ABCC8 variants identified in Spanish pulmonary arterial hypertension patients
Source: Sci Rep. 2020 Sep 15;10:15135. doi: 10.1038/s41598-020-72089-1 (PMC7492224; doi:10.1038/s41598-020-72089-1)

CHARACTERIZATION OF RARE *ABCC8* VARIANTS IDENTIFIED IN SPANISH PULMONARY ARTERIAL HYPERTENSION PATIENTS

Mauro Lago-Docampo[1,2]; Jair Tenorio[3,4,5]; Ignacio González-Hernández[6,7,8], Carmen Pérez-Olivares[7,8,9], Pilar Escribano-Subías[7,8,9]; Guillermo Pousada[2], Adolfo Baloira[10]; Miguel Arenas[1,2]; Pablo Lapunzina[3,4,5]; Diana Valverde[1,2*]

[1] CINBIO, Universidade de Vigo, Vigo, Spain.

[2] Instituto de Investigación Sanitaria Galicia Sur, Hospital Álvaro Cunqueiro, Vigo, Spain.

[3] Instituto de Genética Médica y Molecular (INGEMM), Hospital Universitario La Paz-IdiPaz, Universidad Autónoma de Madrid, Madrid, Spain.

[4] Centro de Investigación Biomédica en Red de enfermedades Raras (CIBERER), Instituto de Salud Carlos III, Madrid, Spain.

[5] ITHACA, European Reference Network on Rare Congenital Malformations and Rare Intellectual Disability.

[6] Servicio de Cardiología, Hospital Universitario Río Hortega, Valladolid, Spain.

[7] Centro de Investigación Biomédica en Red de Enfermedades Cardiovasculares (CIBERCV), Instituto de Salud Carlos III, Madrid, Spain.

[8] Unidad Multidisciplinar de Hipertensión Pulmonar, Servicio de Cardiología, Hospital Universitario 12 de Octubre, Madrid, Spain

[9] Servicio de Cardiología, Hospital 12 de Octubre, Madrid, Spain

[10] Servicio de Neumología, Complejo Hospitalario de Pontevedra, Pontevedra, Spain.

***Corresponding Author**:

Dr. Diana Valverde. Departamento de Bioquímica, Genética e Inmunología. Universidad de Vigo, Campus Universitario As Lagoas-Marcosende s/n, Facultad de Biología, Vigo 36310 (Spain).

Phone number: (+34) 986 811 953 / e-mail: dianaval@uvigo.es

SUPPLEMENTARY INFORMATION

SUPPLEMENTARY TABLE 1. Baseline characteristics of the studied cohort. Data shows absolute number (± refers to standard deviation).

**Baseline characteristics**	**Adult cohort**	**Pediatric cohort**
Age, years	45 (± 0,76)	9.9 (± 4.4)
Female sex	411 (71 %)	27 (60 %)
**Etiology**		
Idiopathic	262 (45.25 %)	23 (51.1 %)
Familial	31 (5.35 %)	4 (8.9 %)
Pulmonary Venooclusive Disease	54 (9.33 %)	3 (6.7 %)
Connective Tissue Disease	91 (15.72 %)	-
Congenital Heart Disease	88 (15.2 %)	11 (24.4 %)
Drugs	22 (3.8 %)	-
Portopulmonary Hypertension	11 (1.9 %)	-
Hereditary Hemorrhagic Telangiectasia	7 (1.21 %)	-
Human Immunodeficiency Virus	13 (2.25 %)	-
Other	-	4 (8.9 %)

**Race**		
White	510 (88.1 %)	39 (86.7 %)
Hispanic	37 (6.4 %)	3 (6.7 %)
Romani	23 (4 %)	3 (6.7 %)
Black	1 (0.2 %)	-
North African	6 (1%)	-
Asian	1 (0.2 %)	-
Hindu	1 (0.2 %)	-

SUPPLEMENTARY TABLE 2. List of primers used for the minigene assay and the site directed mutagenesis. The temperature used to amplify the constructs is also given. F = Forward and R = Reverse, bp = base pairs.

Target	Minigene Construct Primers 5' - 3'	Mutagenesis Primers 5' - 3'	Size (bp)	PCR °C
**c.211C>T p.(His71Tyr)**	F - AAACTCGAGGGGGAGGTACCAGCATAGGA	F - GCTTCATTTCCCTGGGTACAACCTGCGGTGGATCC	674	55
	R - AAAGCTAGCAAGGTAACTCCGGCAGGTTG	R - GGATCCACCGCAGGTTGTACCCAGGGAAATGAAGC		
**c.298G>A p.(Glu100Lys)**	F - CACGAATTCATGGGAGACTAAGAGTACCCAC	F - GCAGATGGTGGGATTTGGTCACCCTGAGATG	1021	60
	R - AAAGCTAGCTTTGCCCACAGTGACATCTT	R - CATCTCAGGGTGACCAAATCCCACCATCTGC		
**c.1429G>A p.(Val477Met)**	F - CCAGAATTCCCCTCTGCTTGCATGAGAG	F - CCTGTCCAGTACTTCATGGCCACCAAGCTG	1173	60
	R - AAAGCTAGCAGCACATAGCAGGCTTTCCA	R - CAGCTTGGTGGCCATGAAGTACTGGACAGG		
**c.1643C>T p.(Thr548Met)**	F - AAAACTCGAGCCTAGCCTACTGGAGCTGTGC	-	403	65
	R - AAAAGCTAGCCTAAGCCTCCGGTCTTCCAG	-		
**c.2422C>A p.(Gln808Lys)**	F - ACAGGATCCCCCAACCAATTCCATCCTTC	F - GAAGCCTGCTCTCTGAAGCCAGACATCGACA	1227	61.5
	R - AAAGCTAGCCCTGCCTCAGTTTCCCTATC	R - TGTCGATGTCTGGCTTCAGAGAGCAGGCTTC		
**c.2694+1G>A**	F - AAAAGCTAGCGACAACGGATTGGTTCCTGCC	-	482	65
	R - AAAACTCGAGGTTGAAGGTCCAGGAGTCAGG	-		
**c.3288_3289del p.(His1097ProfsTer16)**	F - CCAGAATTCCCTGGCAAGATGAATGTGTGTC	F - TGAAGGTGGCCAAGAGACTACCGCAGCCT	1177	60.5
	R - AAAGCTAGCGCTAACCACACACAATGCCT	R - AGGCTGCGGTAGTCTCTTGGCCACCTTCA		
**c.3394G>A p.(Asp1132Asn)**	F - AAAACTCGAGAAATTGGCAGAGGATGCCAGA	-	351	65
	R - AAAAGCTAGCTAATCGGATCGGGGACACTG	-		
**c.3976G>A p.(Glu1326Lys)**	F - ACAGAATTCCCTTCGCAGCCCTTGTGTGTG	F - CCCAGGAGCCCCTTGTAGCTCTCTGCC	1296	63
	R - AAAGCTAGCGTCGGTCTCCTTGGTGGATGAG	R - GGCAGAGAGCTACAAGGGGCTCCTGGG		

SUPPLEMENTARY TABLE 3. List of transcription factors binding in each Genehancer region. All the data is available in the Genecards *ABCC8* entry (https://www.genecards.org/cgi-bin/carddisp.pl?gene=ABCC8).

Genehancer	Transcription Factor Binding sites
**GH11J017401**	-
**GH11J017404**	HDAC1, FOXA2, NFXL1, RAD21, YY1, ZNF766, EGR1, RCOR1, IKZF2, RXRA, REST, NR2F2, SREBF1, SP1, IKZF1, ZBTB33, HNF4A, KDM1A, CTCF, BCOR, NCOA3, ZMYM3, MAX, BACH1, EBF1, ZNF316, CTBP1, POLR2A, HNF4G, NFE2, MAFK, NR2F1, MAFG, MEF2B, GABPA, XRCC5, JUND, ATF3, ZFX, EMSY
**GH11J017411**	ZNF664, ZFHX2, NFIC, ZNF189, NFIB, IKZF1, EGR2
**GH11J017412**	CTCF, RXRA, MAFG, CBFA2T3, RFX5, YY1, SP1, MAFF, ZNF316, CBFA2T2, HNF4A, MAFK, EMSY
**GH11J017432**	ZFHX2, KLF1, EGR2

SUPPLEMENTARY TABLE 4. MaxEntScan of the exons from the minigene constructs. Capital letters represent exonic sequence while lower letters represent intronic sequence.

Variant	First Exon	Donor sequence	Score	Last Exon	Aceptor Sequence	Score	Minigene
**c.211C>T:p.(His71Tyr):**	**2**	TGGGGAAGT	**2.59**	**2**	TGGgtgagt	**8.73**	**worked**
**c.298G>A:p.Glu100Lys**	**3**	agGGTGACC	**2.27**	**3**	CCCAAGCTGCTAATTGgtaggtg	**0.51**	**worked**
**c.1429G>A:p.Val477Met**	**9**	acccatgacccccttcctcagATC	**7.68**	**10**	CCAgtgagt	**8.28**	**worked**
**c.1643C>T:p.(Thr548Met)**	**11**	TCCCTGTGCTTCCTCTGCAGTTT	**10.38**	**11**	ATAGTAAGT	**8.64**	not worked
**c.2422C>A:p.(Gln808Lys)**	**20**	cagGTACAA	**7.09**	**21**	TTGgtgagt	**9.27**	not worked
**c.2694+1G>A**	**22**	ttcctctcccttctgcccagGAT	**8.34**	**22**	TGGgtgag	**6.74**	not worked
**c.3288_3289del:p.(His1097ProfsTer16)**	**24**	aggccacatctgtcttttagGAG	**7.06**	**26**	GAGgtaccc	**7.84**	not worked
**c.3394G>A:p.Asp1132Asn**	**27**	CCTGGTCGATGGTGTTACAGTCA	**4.5**	**27**	AAGGGGCGT	**1.84**	**worked**
**c.3976G>A:p.(Glu1326Lys)**	**31**	cagctcctctccctccccagGAG	**9.78**	**33**	AAGgtcaga	**6.7**	not worked

SUPPLEMENTARY FIGURE 1. UCSC genome browser entry for *ABCC8* focusing on regulatory elements. Genehancer positions are marked with their codes.

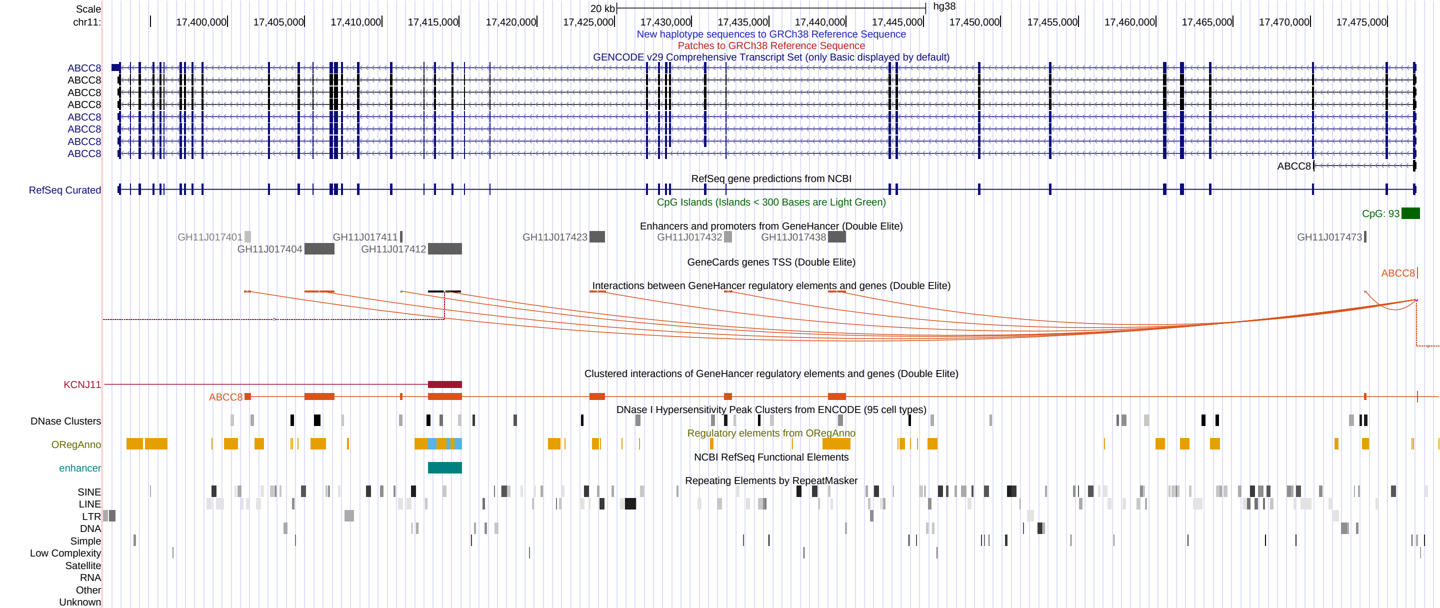
Scale
chr11:
20 kb
hg38
17,400,000
17,405,000
17,410,000
17,415,000
17,420,000
17,425,000
17,430,000
17,435,000
17,440,000
17,445,000
17,450,000
17,455,000
17,460,000
17,465,000
17,470,000
17,475,000
New haplotype sequences to GRCh38 Reference Sequence
Patches to GRCh38 Reference Sequence
GENCODE v29 Comprehensive Transcript Set (only Basic displayed by default)
ABCC8
ABCC8
ABCC8
ABCC8
ABCC8
ABCC8
ABCC8
ABCC8
ABCC8
RefSeq gene predictions from NCBI
RefSeq Curated
CpG Islands (Islands < 300 Bases are Light Green)
CpG: 93
Enhancers and promoters from GeneHancer (Double Elite)
GH11J017401
GH11J017411
GH11J017423
GH11J017432
GH11J017438
GH11J017473
GH11J017404
GH11J017412
GeneCards genes TSS (Double Elite)
ABCC8
Interactions between GeneHancer regulatory elements and genes (Double Elite)
Clustered interactions of GeneHancer regulatory elements and genes (Double Elite)
KCNJ11
ABCC8
DNase I Hypersensitivity Peak Clusters from ENCODE (95 cell types)
DNase Clusters
Regulatory elements from ORegAnno
ORegAnno
NCBI RefSeq Functional Elements
enhancer
Repeating Elements by RepeatMasker
SINE
LINE
LTR
DNA
Simple
Low Complexity
Satellite
RNA
Other
Unknown

SUPPLEMENTARY FIGURE 2. Protein stability analysis of the variants and the original model. Comparison of the stability of the models and the template used to generate them. All the models show less stability than the template 6C3O, expected when comparing simulated data and an experimentally generated template (Gray for skipping models and black for missense).

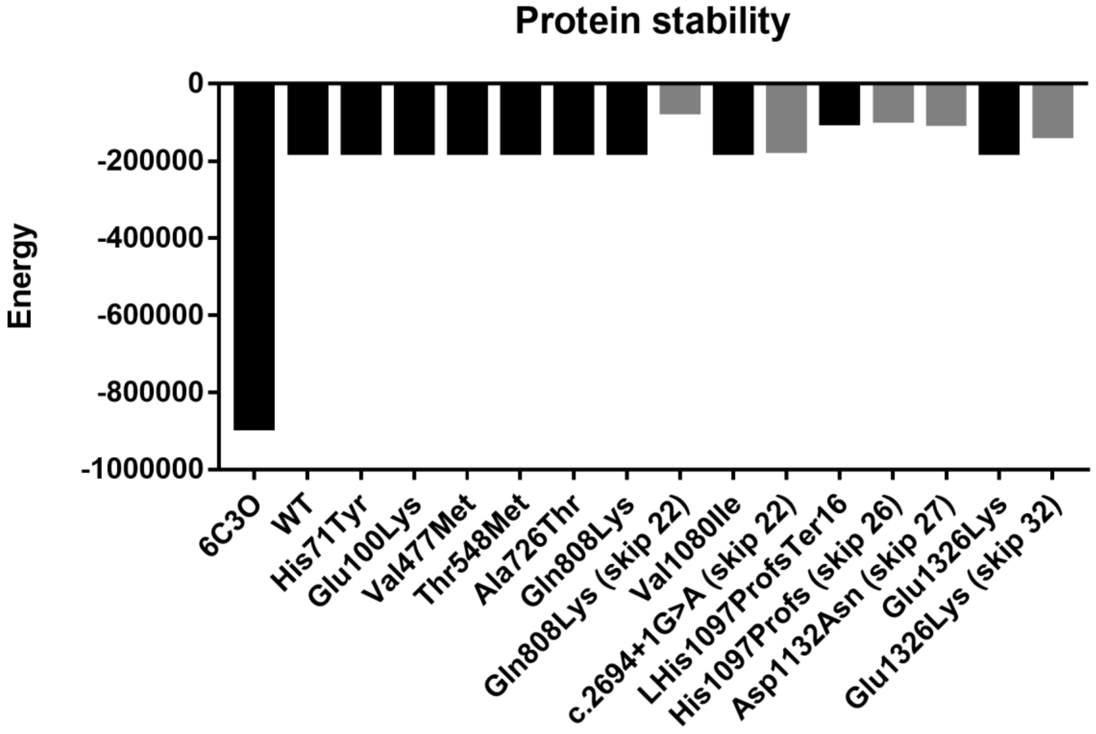

Gels depicted in Figure 2.

Exon 2 minigene wild type and mutated.

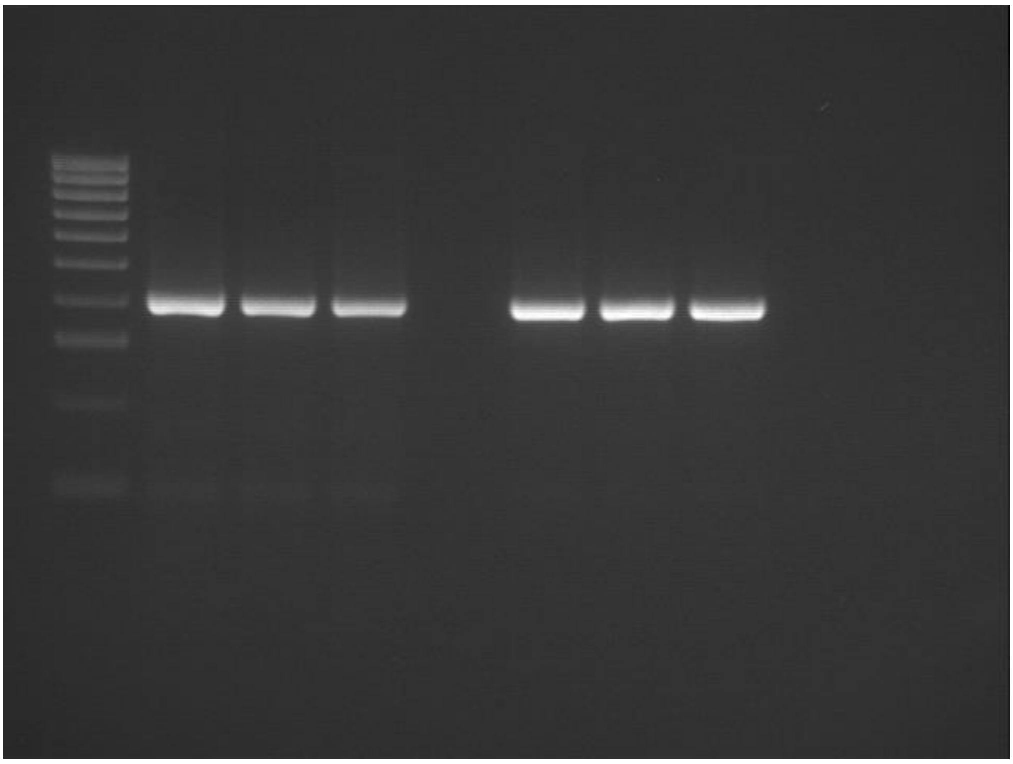

Minigene exon 3 wild type and mutated.

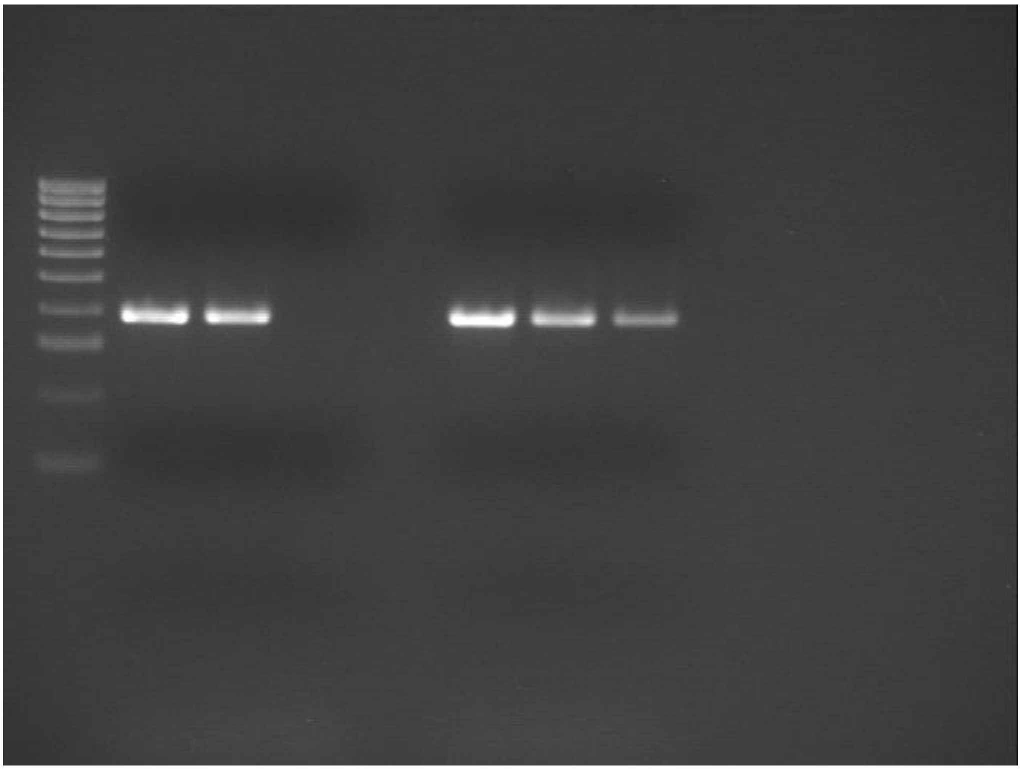

Minigene exons 9 and 10 WT (wells 2, 3 and 4). The wells 6, 7 and 8 correspond to the minigene for exon 20. The band on the right corner corresponds to a degraded DNA ladder.

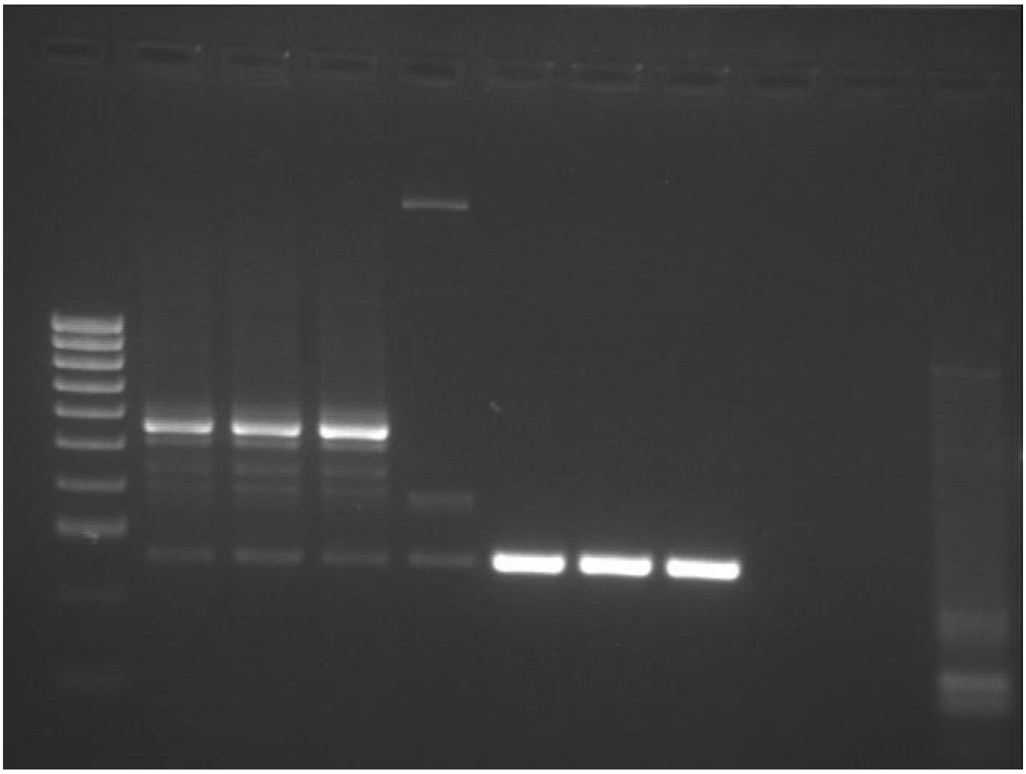

Minigene for exon 9 and 10 after mutagenesis

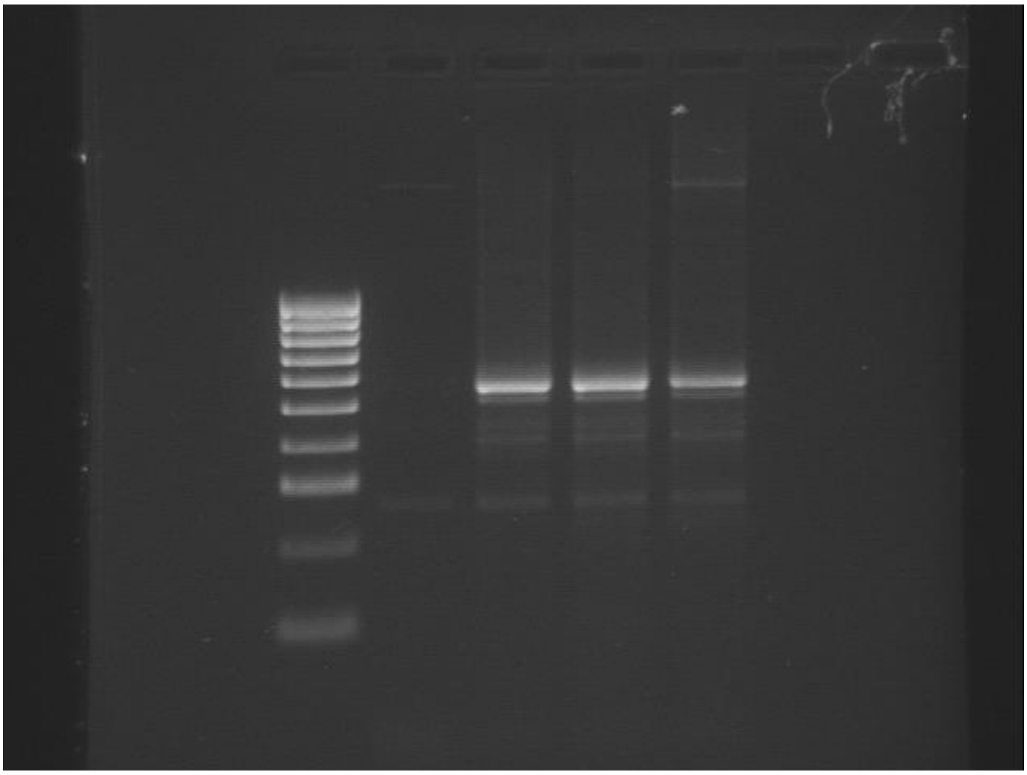

Minigene for exon 27 (right corner), cropped in figure 2.

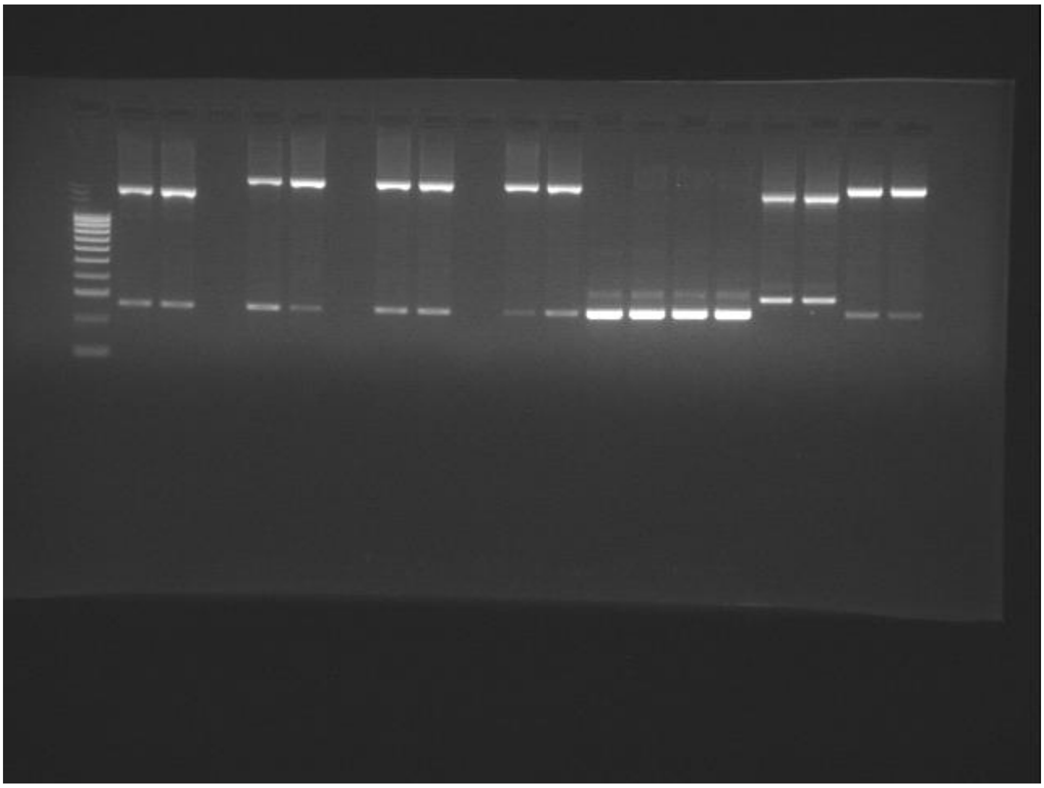

Ilustrative minigene for figure 2E. It shows the minigenes for exon 11 (wild type and mutated) and exon 26 (wild type and mutated) respectively.

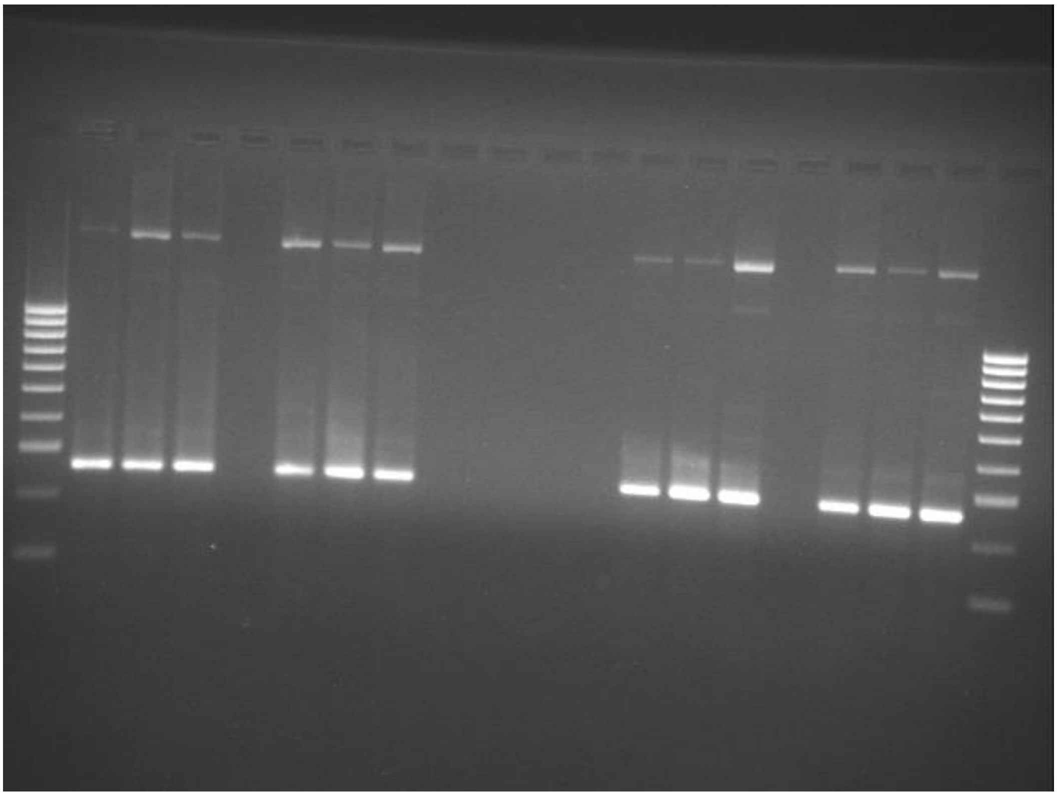

Supplement: Supplementary file 1 — Supplementary Information. [file 41598_2020_72089_MOESM1_ESM.pdf]
